# Paradoxical attitudes toward premarital dating and sexual encounters in Tehran, Iran: a cross-sectional study

**DOI:** 10.1186/s12978-016-0210-4

**Published:** 2016-08-30

**Authors:** Mahnaz Motamedi, Effat Merghati-Khoei, Mohammad Shahbazi, Shahrzad Rahimi-Naghani, Mehrdad Salehi, Mehrdad Karimi, Ahmad Hajebi, Farideh Khalajabadi-Farahani

**Affiliations:** 1 Student Research Center School of Nursing and Midwifery, Isfahan University of Medical Sciences, Isfahan, Iran; 2 Family & Sexual Health Division, Brain & Spinal Cord Injury Research Center (BASIR), Tehran University of Medical Sciences (TUMS), Tehran, Iran; 3 Iranian Center of Addiction Studies (INCAS), Institution of Risk Behaviour Reduction, Tehran University of Medical Sciences (TUMS), Tehran, Iran; 4 Department of Behavioral and Environmental Health, School of Public Health Initiative, Jackson State University, Jackson, MS USA; 5 Institute of Tropical Medicine and International Health, Charité – Universitätsmedizin Berlin, Berlin, Germany; 6 Psychiatry, Department of Psychiatry, Isfahan University of Medical Sciences, Isfahan, Iran; 7 Department of Epidemiology and Biostatistics, School of Public Health, Tehran University of Medical Sciences (TUMS), Tehran, Iran; 8 Psychiatry, Mental Health Research Center, Tehran Institute of Psychiatry- School of Behavioural Sciences and Mental Health, Iran University of Medical Sciences, Tehran, Iran; 9 Department of Population, Health & Family Planning, National Population Studies and Comprehensive Management Institute, Tehran, Iran

**Keywords:** Sexual attitude, Premarital sex, Virginity, Sexual health, Sexual encounters

## Abstract

**Background:**

The purpose of this study is to assess attitudes toward premarital dating and sexual encounters in individuals aged 15–49 years in Tehran.

**Methods:**

Utilizing the attitudes section of an original cross-sectional study (*n* = 755) aimed at assessing sexual health needs of adults, this paper examined personal attitudes towards premarital dating, non-sexual relationships and sexual encounters in both male and female adults aged between 15–49 years. Multi-stage cluster random sampling and a validated/reliable questionnaire were used. Descriptive, bivariate and multivariate analyses were conducted using statistical software.

**Results:**

The results indicated that the majority of the participants were supportive of dating. Almost three-fourths of the males were more positively inclined towards non-sexual, yet tactile, affectionate interactions between unmarried males and females as opposed to only half of the females (70 % vs. 50.5 %). Also, males held significantly more liberal attitudes than females in their acceptance of premarital sex. On preserving virginity prior to marriage, 43 % of the males felt that it was important for a female to be a virgin, whereas only 26 % felt it was important for males to remain a virgin. Interestingly, more females (61 %) supported the importance of a female’s virginity compared with the importance of males’ virginity (48 %). This study showed that, being a male, of a younger age, single, and being less religious or being secular were important determinants of a liberal sexual attitude.

**Conclusion:**

These results might reflect a socio-cultural transition in the sexual attitudes of different age groups of participants - a phenomenon that will need empirical studies to unpack in the Iranian socio-cultural context.

## Background

According to the World Health Organization (WHO) individual sexuality is, to a large extent, determined and influenced by social norms and family values [73]. Hence, sexual attitudes and permissiveness are conceptualized and understood differently according to societal contexts [38, 41]. In fact, social construction of sexuality in any given culture defines sexual behaviours of men and women [22, 38]. Sexual permissiveness entails accepting a wide range of sexual attitudes and associated behaviours, and is influenced by various cultural factors including religious beliefs and the importance of economic exchange at marriage, as well as increased exposure to external influences due to the expansion of worldwide communications and economic changes [38].

There have been significant transformations in sexuality and sexual behaviours in special cultural contexts [36]. Attitudes toward sexuality and its ethical aspects have been altered over the past few decades in many parts of the world [54, 63, 65, 69]. Increasing concerns exist about the consequences of such transformations in conservative and religious societies versus other liberal societies, because empirical research has documented an inverse association between religiosity and liberal sexual attitudes [17]. Iran as a conservative and religious society is no exception.

Similar to other countries in Asia (India, China, Philippines; Thailand; Malaysia, Viet Nam and others), Iran has experienced significant social and attitudinal changes over the past decades [7, 8, 24, 25, 49, 55, 58]. Factors such as westernization, modernization, education, social networks and worldwide communications, information technology; and a rapidly widening generational gap have created grounds for changes in value systems and norms within this country [37, 51, 56, 62].

What perhaps makes Iran, interesting as a case is that since 1979, religion is the core cement of the Iranian value system and norms due to Islamic Law. Dynamic interactions between cultural traditions, religious cultures, and cultural modernity which focuses on “liberal values” are unavoidable [22, 41]. Both religious and non-religious scholars have pointed out that pervasive new norms may change the social structure as well as people’s definitions of sexual behaviour (Aghajanian, Family and Family Change in Iran, forthcoming) [15, 47].

Despite the aforementioned emerging changes, sexual intimacy and sex is only acceptable within the institution of marriage within Iranian society. Premarital sex is considered sinful according to the Islamic religious perspective [5, 30, 61]. It is also legally prohibited and culturally forbidden in this society [35]. Despite the socio-cultural pressure in Iran stressing on marriage as the fundamental core for family formation, the younger generation of Iranian females in particular over the last three decades postpone their marriage [1]. They cite socio-economic reasons for the delay in marriage, or their wish to pursue education [68]. The increasing trend of delayed marriages possibly explains attitude changes toward premarital sexual encounters in the Iranian context.

Postponing marriage has widened the gap between puberty and marriage and this has led to the higher likelihood of people living in metropolitan contexts to be more open, accepting and engaging in premarital heterosexual interactions including sex [29, 32, 33]. However, despite this, in certain settings where only marital sex is documented, this may give the mistaken impression that the age of first sexual intercourse has increased [13].

Sexual behaviours are influenced by a range of factors such as personal attitudes and beliefs, knowledge about sex and its consequences, situational factors, feelings and desires [9, 57]. According to the Theory of Planned Behaviour, an individual’s behaviour is predicted by his/her intentions, attitudes toward the behaviour, perceived social norms and behavioural control [6]. In social psychology, it is widely accepted that attitudes are socially learned [26]. Challenges between traditional and modern values, the advent of technology and its own culture, and the coming of new communication technologies such as social media to people’s life have led to drastic changes in the personal attitudes of Iranians which contributes to changes in previously accepted norms [10, 70].

Increasing premarital heterosexual friendships and dating with the opposite sex among young Iranians has been documented. In a survey among 1,378 unmarried female college students from four universities in Tehran in 2005–2006, 52 % of females reported ever having a boyfriend [32].

Since friendship with the opposite sex is not acceptable in Iranian families, in many cases, particularly for females, families are mostly unaware of any such interactions or relationships. Most parents do not play a key role in informing, educating or supporting their young people on how to manage their friendships and the potential risks posed [39]. Similar to other Islamic states such as Pakistan, in Iran, religiosity is considered to be protective for premarital sex, but it seems that due to the gap between marriage and puberty, and changes in social networks, media and communication technology as well as socio-economic development, the protective role of religiosity is diminishing [11, 27, 56, 62, 64]. Furthermore, there is no comprehensive sexuality education for singles; hence individuals in intimate relationships including sex are unprepared to deal with potential risks for both physical and mental health such as non-consensual sex, sexual coercion and unwanted pregnancy. Undesirable outcomes such as unwanted pregnancy, unsafe abortion, and HIV/AIDS have been associated with premarital sex, particularly among adolescents and young adults [71, 72].

Yet, another issue is that reportedly Iran has entered into its third wave of an HIV epidemic, which is one of the most serious health risk issues in the country. Although the HIV epidemic is primarily concentrated among key populations such as injecting drug users, HIV transmission through unsafe sex is on rise among adolescent and young adults, with recent studies suggesting an increase in premarital sexual encounters in Iran [4, 11, 20, 33]. The main feature of this peak is the significant shift in new cases of HIV infection from intravenous drug use to unsafe sexual practices. Since 1988 till 2005, from among all HIV positive cases (30,183 cases), 67 % was due to injecting drug use and 18 % was because of unsafe sex, while the corresponding rates among HIV positive cases in 2014, was 41 % and 36 %, respectively. These rates show a significant rise in sexual transmission of HIV in Iran [45].

It is within the interest of the current global health context that this empirical study, focusing on attitudes toward premarital dating and sexual encounters in Iran is both, socioculturally relevant in order to highlight shifting attitudes and perceptions as it is from the point of better elucidating gaps in current public health practices. The purpose of this study is to assess attitudes toward premarital dating and sexual encounters among adults aged 15-49 years in Iran.

### Conceptual framework

Studies among female college students have shown that young people believe that social norms are still against premarital sex in Iran [31, 40]. In contrast, evidence indicates a rise in premarital sex, particularly among young men and women (aged 18–34) in metropolitan cities of Iran [59]. This contrast persuaded a team of researchers to hypothesize that changes in sexuality in the Iranian contexts might be related to changes in personal attitudes, rather than social norms which are more resistant to change. Social norms influence personal attitudes and behaviors, although the links are complicated and may be bi-directional [19]. Many factors affect personal attitudes towards premarital sex, including perceived norms and an individual’s own sexual experiences [9, 38]. However, in this study, the associations between sexual attitudes and age, biological sex, education, religiosity and marital status have been sought out.

## Methods

This paper is based on the quantitative data of an original mixed methods study. This cross sectional study was conducted from March 2014 to May 2015. Questionnaires were administered to 800 males and females aged 15–49 years residing in Tehran, the capital of Iran. These individuals were presented with a consent form to sign. Seven hundred and fifty five individuals completed the questionnaire. The participants included 410 women (56%) and 344 men (46%). Forty five people decided to withdraw from completing the study questionnaire. About 12 million people live in the city of Tehran [67], which is a large metropolis with different cultures and ethnic groups. Given this, the residents are more influenced by modern ideas and social changes than those in small towns and villages.

To obtain a representative sample of adults in Tehran, the city was divided into three regions based on the population density; region with large, medium- and small-population. Each region comprised of several districts. According to the proportion of population to the total population in each district, the required sample was calculated in each section.

Applying Cochran’s formula for estimating sample size, the sample required was calculated as 768 participants (the proportion of sample population size for each region; the large, medium, and small population region was 422, 249, and 97, respectively). Then a district from each region was randomly selected. These districts house a number of community health centers, public parks and public places such as venues for cultural activities. From each district, one community health center, one venue for the cultural activities, and one public park was randomly selected. Upon the ethics committees’ approval (by the Ethics Committee in Tehran University of Medical Science as well as by the Isfahan University of Medical Sciences), trained staff utilized a convenience sampling approach (at different times of day; morning and evening) and recruited qualified individuals to participate. Trained staff explained the objectives of the study to the individuals who agreed to complete the questionnaire. Female staff assisted female participants to complete the questionnaires and male staff assisted the male participants. Same sex interviewers were believed to enhance response rate because of cultural sensitivity of this topic which was strength of this study. Each participant spent 30–45 mins to complete the questionnaire. Only eligible volunteers were recruited for this study. No incentives were provided for participation in this study.

The survey instrument was adapted from the World Health Organization (WHO) questionnaire for assessing sexual and reproductive health of young people [14]. This questionnaire was designed in order to assess knowledge, attitudes, behaviors and sexual health outcomes. Back-translation from English version of questionnaire to Persian was initially conducted blindly by two independent bilingual (English and Persian) experts and validated in a separate study by the research team [43]. Content and face validity and reliability of the questionnaire was assessed and confirmed by both qualitative and quantitative methods for the age group 15–49 years and both single and married people. The questionnaire was edited to be suitable as a self-administered questionnaire.

The dependent variable reported in this paper is a scale variable named “personal attitude toward premarital sex”. This scale was constructed by sum of scores of nine aligned items or statements about the acceptability of a range of premarital intimacy situations and sex for unmarried men and women (Table 3). Each items was measured by three-point Likert scale (1- agree, 2- not sure, 3-disagree). The score of attitude scale ranged from 9 to 27 (Cronbach ‘Alpha = 0.80). The higher the score, the more liberal is the attitude towards premarital sex and vice versa. Independent variables included age, sex, education, marital status and religiosity. The sample was also weighted at the stage of analysis to make sure it was similar to the population age structure according to Iran’s 2011 census.

Firstly, socio-economic status and responses to attitudinal statements were described. To compare the attitudinal scale scores by independent variables such as age, educational level, biological sex, marital status and religiosity, *t*-Test and ANOVA were applied. Finally, those factors significantly associated with personal attitudes and were not highly correlated, were entered into multivariate analysis (linear regression) to detect determinants of liberal personal attitudes toward premarital sex. In this study *P*-values less than 0.05 were considered significant.

## Results

### Socio-economic and demographic characteristics

The mean age of participants in this study was 29.4 years (standard deviation: 8.30). About 51 % of the participants were never married. One fourth (24 %) had a diploma, and 64.5 % had an academic education. From the latter, 15 % had 2 years of university education, and 48.5 % had a bachelor or higher degree. In response to the question: How important is the role of religion in the choices you make in your life? About one fourth (26 %) of the participants proclaimed “very important”, almost half (47 %) proclaimed “important” and the remaining fourth (27 %) proclaimed that is was not important Table 1.

**Table 1:** Socio-demographic characteristics of the Participants

Characteristics	N (%)
Gender	
Male	345 (45.5)
Female	410 (54.5)
Age Group	
15–24	231 (30.6)
25–33	331 (43.8)
≥ 35	193 (25.6)
Level of Education	
< Diploma	89 (11.1)
Diploma	189 (24.3)
Collegiate	477 (64.5)
Marital status	
Never married	385 (51.1)
Ever married	370 (48.9)
Religiosity	
Very important	199 (26.3)
Important	355 (46.9)
No important	202 (26.7)

### Personal attitudes toward dating and non-sexual interactions and relationships (kissing, touching, hugging)

The results showed that the majority of both men and women (81 % & 82 %) were supportive of dating, i.e. social appointments with the opposite sex in an attempt to get to know each other [74], before marriage; there was no sex difference. In contrast, when it came to other intimate, but non-sexual, tactile interactions and relationships (like hugging, touching and kissing); males were more open to such interactions and relationships than females (69 % vs. 50.5 %; *P* < 0.001).

### Personal attitudes towards premarital sex under different circumstances for unmarried men and women

Again more males than females were supportive of premarital sex if couples “loved one another” (42 % versus. 33 %), although a significant proportion of the participants were particularly skeptical in this regard, given its interpretative subjectivity. In contrast, a greater percentage of women believed that “love” is a precondition for initiating sex than men (80 % versus. 43 %, *P* < 0.001), (Table 2).

**Table 2:** Personal Attitudes of Men and Women Aged 15–49 Years toward Premarital Sex by Gender

Statements	Men (*N* = 344)			Women (*N* = 411)			*p*-value
	Agree (%)	No sure (%)	Disagree (%)	Agree (%)	No sure (%)	Disagree (%)	
Dating and Non-Sexual Relationships Before Marriage							
− I believe it’s all right for unmarried boys and girls to have dates	80.8	9.6	9.6	82.0	6.6	11.4	0.249
− I believe it’s all right for boys and girls to kiss, hug and touch each other	69.9	10.7	19.4	50.5	11.2	38.3	0.000
Acceptability of Premarital sex Under different Circumstances for Unmarried Men and Women							
− I believe there is nothing wrong with unmarried boys and girls having sexual intercourse if they love each other	41.7	18.9	34.0	32.6	12.2	52.2	0.000
− A boy and a girl should have sex before they become engaged to see whether they are suited to each other	43.3	22.7	34.0	24.1	16.1	59.9	0.000
− It’s all right for boys and girls to have sex with each other provided that they use methods to stop pregnancy	44.8	22.1	33.1	20.9	17.0	62.0	0.000
− I think that you should be in love with someone before having sex with him/her.	42.9	13.3	43.5	79.9	37.0	11.2	0.000
− One night stands are OK	45.9	18.0	36.0	7.3	12.1	80.6	0.000
Importance of Virginity for Unmarried Men and Women							
− I believe that girls should remain virgins until they marry	43.0	30.2	26.7	61.2	17.5	21.4	0 .000
− I believe that boys should remain virgins until they marry	26.2	15.1	58.7	48.4	18.7	32.8	0.000

A smaller percentage of the participants were supportive of sex before marriage to ensure sexual compatibility after marriage. This percentage among males was statistically almost double to that of females (43 % versus. 24 %). A higher percentage (34 % of males and 60 % females) were against premarital sex and a significant proportion was unsure about their stance in this regard (23 % of males and 16 % females). Forty five percent of males and 21 % of females agreed with sexual relations between males and females provided that they used methods to prevent pregnancy, but the percentage who disagreed with any sort of sexual contact was 33 % and 62 % in men and women, respectively (*P* < 0.001).

### Attitudes towards the importance of preserving virginity for never-married men and women

Males were significantly (43 %) less likely to insist upon the necessity to preserve the virginity of girls/women until marriage, than were females (61 %). This means that women are more concerned about protecting virginity until marriage than men are. Uncertainty about the importance of preserving a girl’s virginity issue was significantly higher for men (30 % of men and 17.5 % women) (*P* < 0.001). While, only one fourth of the males were supportive of males abstaining from sex before marriage (i.e. men should remain virgins until marriage), a greater percentage of women (about 50 %) also believed that men should abstain from sex or remain virgins until marriage (*P* < 0.001).

### Bivariate analysis

To identify factors that are associated with liberal sexual attitudes, the mean score of the attitude scale toward premarital sexual behavior was compared between categories of independent variables (biological sex, age, educational level, religiosity and marital status) by *t*-test and ANOVA. Results show that mean score of attitudes being open towards premarital sexual behaviours was significantly lower among women than that of men (*P* < 0.001). The difference is significant, but small. Younger people (age group 15–24) held more liberal sexual attitudes than people aged between 35–49 years (mean score 19.55 vs. 17.39, *P* < 0.001). Never married participants had more liberal sexual attitudes than ever married respondents (20 vs. 18, *P* < 0.001). The attitudes of men and women toward premarital sex were not significantly associated with educational levels. Finally, those who were more religious (those who reported religion is very important or important in their life) held more conservative attitudes towards premarital sex compared to those who were less religious (those who reported religion is not important in their life), (mean score 18.32 vs. 22.44, *P* < 0.001) (Table 3).

**Table 3:** Mean Scores of Attitudes toward Sexual Permissiveness by Selected Social and Demographic characteristic

Characteristics	N (%)	Sexual Attitude (9-27)	*P*-value
Gender			
Female	345 (45.5)	17.64	<0.001
Male	410 (54.5)	17.70	
Age (years)			
15-24	231 (30.6)	19.55	
25-34	331 (43.8)	19.14	<0.001
35-49	193 (25.6)	17.39	
Marriage status			
never married	385 (51.1)	19.94	
ever Married	370 (48.9)	17.55	<0.001
Level of Education			
<diploma	89 (11.1)	18.38	
Diploma-technical	189 (24.3)	18.75	= 0.566
Collegiate	477 (64.5)	18.47	
Religiosity			
Very important	199 (26.3)	15.35	
Important	355 (46.9)	18.32	<0.001
No important	202 (26.7)	22.44	

### Multivariate analysis

Multivariate analysis of factors associated with sexual attitudes is shown in Table 4. Accordingly, the mean score of attitudes decreases by 1.12 when the gender increase one unit (become females from males) (*P* < 0.001). This means that sexual attitudes among men are more liberal than women. The mean score of attitudes among people aged 15–24 and aged 25–34 years increases by 0.816 and 0.794, compared to the mean score of attitude among people aged 35 and older, respectively. This means that the younger generation are more liberal in their sexual attitudes than older generation (*P* < 0.05 and *P* < 0.001). The mean score of attitudes increases by 0.804 when marital status increases one unit (married changes to unmarried), which means unmarried people are more liberal in sexual attitudes than married ones (*P* < 0.001). Finally, multivariate analysis showed that the mean score of attitudes among people who were either very religious or were religious decreases by 6.49 and 3.67 compared to the scores among non religious people (*P* < 0.001). This means that religious people had significantly greater conservative sexual attitudes than nonreligious people. Interestingly, education did not appear to be a significant determinant of sexual attitudes after controlling for other factors. Hence, this study shows that being a male, of a younger age, single, and being less religious or secular are important determinants of the liberal sexual attitude after controlling for other factors.

**Table 4:** Multivariate analysis of factors associated with Attitudes toward Sexuality before Marriage among people aged 15–49 (*N* = 755)

Variable	B	SE	*P*-value
Gender			
F	-1.122	0.206	<0.001
M (ref)	0		
Age			
15–24	0.816	0.347	<0.05
25–34	0 .794	0.238	<0.001
> =35 (ref)	0		
Level of Education			
< diploma	0.014	0.323	0.966
Diploma-technical	0.397	0.237	0.095
collegiate(ref)	0		
Marriage status			
Never married	0.804	0.248	<0.001
Ever married(ref)	0		
Religiosity			
Very important	-6.495	0.283	<0.000
Important	-3.667	0.253	<0.000
No important (ref)	0		

## Discussion

### Signs of changes of sexual attitudes by time over the past decades

Comparing personal attitudes towards premarital sex shown in this study with those in previous studies, indicates some attitudinal changes in recent decades. In the past, sexual attitudes were more restrictive than they were during the time of this study. Studies published about sexual attitudes and practices before 2000 are very scarce in Iran. An unpublished report of one such study in 1993, among 18,000 Iranian students aged 15–18 years indicated that only 17 % of boys and 6 % of girls were supportive of premarital relationship between unmarried boys and girls (Mohammad, Attitude regarding relationship between boys and girls among college students, unpublished). In 2004, the National Survey of the Attitudes and Values of Iranians among 4,581 men and women (mean age = 32.2 years) showed that only 38 % of participants were supportive of premarital heterosexual relationships, 12 % were uncertain, and 50 % were against such relations [44]. In another study conducted in 2002 among 1385 adolescent boys in Tehran, 55 % had a liberal attitude towards premarital friendships with the opposite sex. Only 28.5 % of adolescent males had a liberal attitude towards premarital sex for men and 15.5 % had a liberal attitude towards premarital sex for women [46].

Comparing the above mentioned rates with those of the current study suggests greater openness toward premarital relationships including sexuality in the latter. The majority of those surveyed were in favour of premarital dating (81 % of boys and 82 % of girls) and even more liberal in attitudes towards premarital sex. Sixty seven percent were supportive of any type of sexual contact before marriage for men and 38 % were supportive of such relations for women. Forty two percent of men and thirty three percent of women were supportive of premarital sexual intercourse for unmarried girls and boys if the relationship was romantic. These differences might be indicative of some attitudinal transformation, which could lead to further change of norms on sexuality in the future in Iran ([11, 21, 62]). Because of the close relationship between attitudes and behaviours, these results need to be considered in further examining of sexual interactions and behaviours among young people before marriage in the country.

Comparing these results with what people perceive as sexual norms also suggests that people are more liberal in their sexual attitudes than their perception of social norms of sexuality before marriage [33, 40, 51]. These changes in attitudes can also influence perceived norms in the future. Another finding which supports possible changes in attitudes toward premarital sex is greater liberal attitudes among the younger cohort in this study than in the older cohort. These findings are consistent with previous studies from other societies [60, 63, 65, 69]).

### Contrast between acceptability of premarital dating and premarital sex

This study showed that premarital dating was somewhat more acceptable than sexual encounters. Of course, opposite sex dating is more likely to create circumstances leading to further intimacy and even sexual interaction. However, in societies like Iran where emphasis is placed on preserving a women’s virginity, heterosexual relationships and dating are socially accepted only with the condition of no sexual contact. This can be a distinct feature of premarital liaisons within conservative societies which have experienced some degree of modernity.

Notably, while the acceptability of premarital dating was reported with less uncertainty (6.6 % to 11.2 %), the acceptability of sex before marriage was accompanied with greater uncertainties. There are possible explanations for these uncertainties, for example, those who were uncertain in answering these questions might have understood sexual encounters differently,

Consistent with our findings, uncertainty was prominent in another study in Delhi, India - more than 40 % of respondents were uncertain about both premarital dating and sex before marriage [34].

### Men indicate more liberal attitudes toward premarital sexual encounters than women

Apart from dating, for all other statements of premarital intimacy including sex, men were more liberal than women. Their greater acceptability of premarital sex may be due to their greater freedom to be involved in such relations than women, in the Iranian context. Gender differences in sexual attitudes and behaviours have also been shown to be true in other societies to some degree [2, 25, 53, 66]. For instance, a study in the United States, which is among the most religious societies in the industrial world, and at the same time, is among the most secular countries [52], reported that women were more conservative in sexual attitudes than men. Moreover, women were less likely to approve of sex outside marriage as well as legal access to pornography than men [65]. Such gender differences in a conservative cultural context could lead to the sexual exploitation of women and holding them responsible if sexual coercion occurs. [23, 42].

### Importance of virginity for women from the perspectives of men and women

In this study, men were more liberal in accepting premarital sex under different circumstances (i.e. in a love and romantic relationship, when contraception is used, to identify suitability for marriage) than women. They were even were less supportive of virginity for women than the females participants. While, 61.2 % of women believed that women should remain virgins until marriage, only 43 % of men held this belief. Hence, men believe they could be sexually experienced before marriage but their marriage partners should be virgins [40]. This might be biased reporting by men but is in line with findings from other studies [2, 12, 25, 51, 53, 66].

### Sex differences in perspectives on circumstances of premarital sex

This study shows that more men favour sex occurring in the context of contraceptive use than women (45 % versus. 21 %). However, a greater number of women consider “being in love” as a precondition for having sex compared to men (80 % versus. 43 %). These results could reflect different rationales and motivations for sex among men and women. A study among 2566 students from five universities in Tehran and Tabriz showed different reasons for premarital sex among men and women. Men were more driven by pleasure, recreation and peer pressure and impulsivity, while females had more emotional reasons for premarital sex [3]. It seems that men are more concerned about the risks of pregnancy, whereas women are thinking more of true love as a condition for sex. These results reflect different perceptions of consequences of sex for men and women. This difference is also partly because females see preserving virginity before marriage as more important than males [11, 28].

These results which highlight sex differences in sexuality before marriage in Iran, can be due to a strong gender double standard. Men tend not to be worried about virginity, partly because they are perceived as being less able to control their sexual desires, and also partly because of not having physical hymen, which is a (often a false) marker of virginity preservation [3, 28]. These sex differences lead to more sexual experience among men before marriage, as well as greater risk taking sexual behaviors such as earlier sexual debut and having multiple partners [28].

### Determinants of liberal sexual attitudes

The results of our study indicate an inverse correlation between religiosity and liberal sexual attitudes. Many previous studies have also shown that sexual permissiveness and sexual practice is significantly uncommon among religious people around the world [18]. Consistent with these results, the majority of studies indicated a reverse relationship between religiosity and premarital sex [16] and number of sexual partners [46, 50]. We must note though that in cross-sectional studies such as ours, causal relationships are difficult to ascertain, because, for instance, religious individuals engaged in sexual relations might reassess their religious values and perceive themselves as a nonreligious individuals.

Never married respondents had more liberal attitudes than ever married participants. This cannot be due to the younger age of single participants, because after controlling the effect of age, marital status remained as a determinant of the attitude. Married respondents might consider greater negative consequences of such relations on marriage compared to unmarried ones [30, 48].

This study is a cross-sectional study and any causal interpretations should, be made with caution. Further, the study population (men and women aged 15–49 years residing in Tehran, the capital of Iran) is not representative of the whole population of Iran. Tehran is a modern and metropolitan city which is a vanguard to social changes and new ideas, these results are more indicative of people in Tehran than the whole country. Sexual attitudes of people across the country as a whole can be expected to be more conservative than these findings [33]. Moreover, due to the sensitive nature of this study and the questions of the instrument, household based sampling was not feasible and the sample was recruited through cultural centers, hence people who attend these centers might be different in terms of education and social attitudes with the whole population. Another shortfall of this study is associated with the limitations to use explicit terms for different types of sexual relationships in the questionnaire. Since, previous research indicated that non-vaginal sex is common to preserve virginity (oral and anal sex), hence that would be ideal to ask about acceptability of vaginal penetrative sex versus non vaginal penetrative sex and non-penetrative sex. This is a limitation of this research which needs to be considered in interpretation of results. It would have been useful, if a question had been included to seek information about their participation in premarital compulsory courses about reproductive health.

## Conclusions

Although some changes are seen in attitudes on the importance of preserving virginity for women prior to marriage, it appears that virginity is still the driver of conservative attitudes towards sexual encounters before marriage in Iranian culture. These findings highlight the importance of considering the future sexual behaviour of young people in Iran. The results of our study need to be considered in assessing sexual behaviour among younger generations and in addressing their sexual health. As sexual attitudes are closely associated with one’s sexuality-related experiences, more research is needed to explore factors affecting sexual attitudes such as access to social networks, media, peers, and family. Qualitative studies about reasons for supporting or opposing premarital sex among men and women and virginity would be useful in this context.

### Recommendations

Information obtained from this study can provide evidence for policy makers and programme managers to inform policies and programmes. The findings from this study can also help the civil society to identify evidence-based needs by utilizing such empirical findings to help the policy makers develop and offer practical solutions for achieving the goals of sexual health awareness.
